# The Impact of Exercise Capacity on Complex Neuromuscular Adaptations: A Narrative Review Based on a Rat Model System Selectively Bred for Low and High Response to Training

**DOI:** 10.1002/cph4.70029

**Published:** 2025-07-28

**Authors:** Vinicius Guzzoni, Upasana Shrestha, Nicholas J. Kesler, Aditya Acharya, Samantha J. McKee, Tatiana Sousa Cunha, Dulce Elena Casarini, Steven T. Haller, David J. Kennedy, Steven L. Britton, Lauren Gerard Koch

**Affiliations:** ^1^ Department of Medicine, School of Medicine Federal University of São Paulo (UNIFESP) São Paulo Brazil; ^2^ Department of Medicine The University of Toledo College of Medicine and Life Sciences Toledo Ohio USA; ^3^ Department of Physiology and Pharmacology The University of Toledo College of Medicine and Life Sciences Toledo Ohio USA; ^4^ Institute of Science and Technology, Department of Science and Technology Federal University of São Paulo (UNIFESP) São José dos Campos Brazil; ^5^ Department of Medicine, Nephrology Division, School of Medicine Federal University of São Paulo (UNIFESP) São Paulo São Paulo Brazil; ^6^ Department of Anesthesiology, Michigan Medicine University of Michigan Ann Arbor Michigan USA

**Keywords:** aerobic exercise, central nervous system, exercise‐resistant phenotype, intrinsic running capacity, skeletal muscle

## Abstract

There is scientific evidence that supports the association between aerobic exercise capacity and the risk of developing complex metabolic diseases. The factors that determine aerobic capacity can be categorized into two groups: intrinsic and extrinsic components. While exercise capacity is influenced by both the intrinsic fitness levels of an organism and the extrinsic factors that emerge during training, physiological adaptations to exercise training can differ significantly among individuals. The interplay between intrinsic and acquired exercise capacities represents an obstacle to recognizing the exact mechanisms connecting aerobic exercise capacity and human health. Despite robust clinical associations between disease and a sedentary state or condition, the precise causative links between aerobic exercise capacity and disease susceptibility are yet to be fully uncovered. To provide clues into the intricacies of poor aerobic metabolism in an exercise‐resistant phenotype, over two decades ago a novel rat model system was developed through two‐way artificial selection and raised the question of whether large genetic differences in training responsiveness would bring about aberrant systemic disorders and closely regulate the risk factors in health and diseases. Genetically heterogeneous outbred (N/NIH) rats were used as a founder population to develop contrasting animal models of high versus low intrinsic running capacity (HCR vs. LCR) and high versus low responsiveness to endurance training (HRT vs. LRT). The underlying hypothesis was that variation in capacity for energy transfer is the central mechanistic determinant of the divide between complex disease and health. The use of the outbred, genetically heterogeneous rat models for exercise capacity aims to capture the genetic complexity of complex diseases and mimic the diversity of exercise traits among humans. Accumulating evidence indicates that epigenetic markers may facilitate the transmission of effects from exercise and diet to subsequent generations, implying that both exercise and diet have transgenerational effects on health and fitness. The process of selective breeding based on the acquired change in maximal running distance achieved during a treadmill‐running tests before and after 8 weeks of training generated rat models of high response to training (HRT) and low response to training (LRT). In an untrained state, both LRT and HRT rats exhibit comparable levels of exercise capacity and show no major differences in cardiorespiratory fitness (maximal oxygen consumption, VO_2max_). However, after training, the HRT rats demonstrate significant improvements in running distance, VO_2max_, as well as other classic markers of cardiorespiratory fitness. The LRT rats, on the other hand, show no gain in running distance or VO_2max_ upon completing the same training regime. The purpose of this article is to provide an overview of studies using LRT and HRT models with a focus on differences in neuromuscular adaptations. This review also summarizes the involved molecular and cellular signaling pathways underlying skeletal muscle adaptations in LRT models in comparison to the HRT model, which responds positively to endurance training. The LRT‐related adverse effects in neuromuscular responses seem to be primarily driven by: (i) impaired glucose tolerance or impaired insulin sensitivity, (ii) increased extracellular matrix (ECM) remodeling, (iii) loss of type I muscle fibers, (iv) mitochondrial dysfunction, and (v) intricate cellular signaling orchestrated by TGF‐ß1‐JNK and TNF‐α‐MAPK pathways. Alternatively, the HRT model demonstrates improved neurovascular and muscle remodeling responses and increased central nervous system excitability, which might reflect an inherent protective mechanism to stress events.

## Graphical abstract

Integrative cellular signaling triggered by the impaired glucose tolerance or impaired insulin sensitivity, mitochondrial dysfunction, mediated by an intricate cellular signaling orchestrated by TGF‐ß1‐JNK and TNF‐α‐MAPK increased extracellular matrix (ECM) remodeling pathways, and loss of type I muscle fibers in LRT models. Adapted from Lessard et al. (2013).

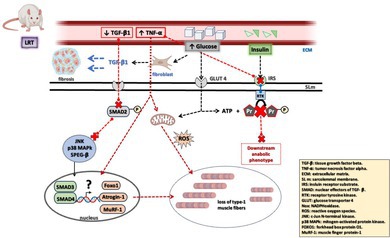

## 1 Introduction

Physical activity offers abundant protection against the deleterious effects of sedentary behavior on whole‐body metabolism (Zhang et al. 2017; Smith et al. 2023) while aerobic exercise training is an effective prescription for ameliorating a wide range of clinical conditions and health outcomes (Pedersen and Saltin 2006; Smith et al. 2023). Resistance training is also an integral part of physical activity guidelines and is associated with improved strength and muscular mass, cardiorespiratory fitness, and a broad spectrum of health outcomes (Steele et al. 2017). Following a long line of investigation, several pieces of evidence underpin the robust association between low intrinsic exercise capacity and a higher probability of developing complex metabolic diseases (Koch and Britton 2018; Mandsager et al. 2018). Recent methodological advances have allowed the field of exercise physiology to progress towards comprehensive systems‐level profiling of the complex molecular interplay that occurs with exercise. These advancements have enabled a better mechanistic understanding of the cause‐and‐effect relationships underlying exercise adaptation and associated health benefits (Smith et al. 2023). The response to exercise training is a multifaceted phenomenon, shaped by a complex interplay of genetic and environmental factors (Steele et al. 2017; Giles et al. 2021). This complexity poses challenges in pinpointing the exact mechanisms that link exercise to the health benefits. Genetic factors, which influence aerobic capacity (maximal oxygen consumption, VO_2max_) and disease risk, interact with environmental factors (e.g., physical training, nutrition) and can be classified into two categories: intrinsic and acquired components. The intrinsic component dictates an individual's baseline aerobic capacity and disease risk profile in the so‐called “sedentary” (nontrained) state. In contrast, the acquired component determines how individuals respond to physical activities, particularly when engaging in regular endurance training (Koch and Britton 2008). While exercise capacity is influenced by both intrinsic fitness levels of an organism and extrinsic factors that emerge during training (Koch et al. 2013), physiological adaptations to exercise training can differ widely among individuals (Farrash et al. 2021). Accordingly, the interplay between intrinsic and acquired exercise capacities, both of which have high inter‐individual variability (Bouchard et al. 1999; Bouchard and Rankinen 2001; Kelahmetoglu et al. 2020), represents an obstacle to identifying the exact mechanisms connecting aerobic exercise capacity and human health. Despite robust clinical associations between metabolic diseases and a sedentary state, the precise causative or mechanistic links between low intrinsic aerobic capacity and disease susceptibility are yet to be fully uncovered.

In an attempt to provide clues into the intricacies of poor metabolism in exercise‐resistant phenotypes, in addition to providing mechanistic detail in contrasting polygenic models, Koch and Britton began to exploit new theoretically based experimental approaches and raised the question of whether large differences in aerobic exercise capacity would bring about aberrant systemic disorders that closely regulate the risk factors for diseases (Koch and Britton 2008). According to their Energy Transfer Hypothesis, variation in capacity for aerobic metabolism, typically measured through maximal oxygen consumption (VO_2max_) is the central mechanistic determinant underlying the segregation between complex disease and health (Koch and Britton 2018). In this sense, genetically heterogeneous outbred rats were bred using bidirectional artificial selection to develop models of high versus low intrinsic running capacity or high versus low responsiveness to endurance training (Koch and Britton 2001, 2019; Koch et al. 2012, 2013). High‐capacity runners (HCRs) exhibit characteristics similar to endurance‐trained individuals, while low‐capacity runners (LCRs) demonstrate a markedly increased disease risk profile, including significantly poorer cardiovascular function (Wisløff et al. 2005; Lujan et al. 2006), hepatic steatosis (Thyfault et al. 2009), reduced peripheral insulin sensitivity and oxidative capacity (Kivelä et al. 2010; Rivas et al. 2011), and shorter life expectancy (Koch et al. 2011).

**FIGURE 1:**
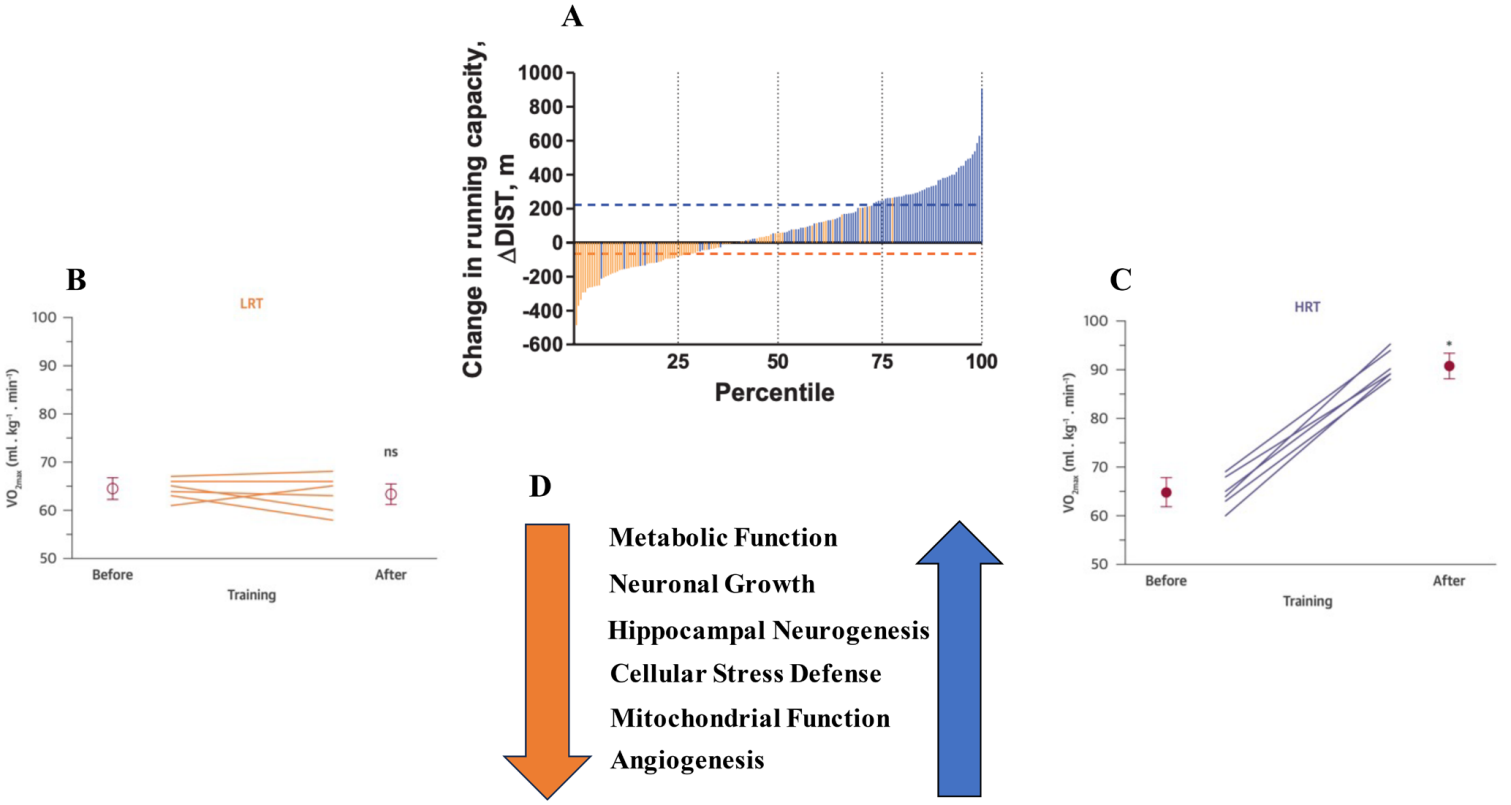
Percentile rank score for the magnitude of change in running capacity (∆DIST) between the low response trainers (LRT) and high response trainers (HRT) rat lines. Orange bars indicate LRT rats, and blue bars indicate HRT rats. Dotted lines indicate the mean change in running capacity for the LRT (orange) and HRT (blue) selected lines (A). Maximal oxygen consumption (VO_2max_) measured before and after high‐intensity aerobic interval training in LRT (B) and HRT. VO_2max_ greatly elevated in the HRT line (C). The adaptational response in the LRT and HRT rats observed across studies (D). Adapted from Koch et al. (2013) and Wisløff et al. (2015).

As another test of their theory, artificial selection based on the acquired component of exercise capacity generated a second contrasting rat model of high‐response‐to‐training (HRT) and low‐response‐to‐training (LRT) rat lines (Koch et al. 2013; Ross et al. 2019). These lines show no differences in their intrinsic (baseline) running capacity but respond significantly different to a standardized regimen of endurance training. The development and use of the outbred, genetically heterogeneous rat models for exercise capacity aims to capture the complexity of disease risk and the diversity of health among humans. Further, accumulating evidence indicates that epigenetic markers may facilitate the transmission of effects of exercise and diet to subsequent generation, suggesting that exercise and diet environments can have a transgenerational effects on health and fitness (Kelahmetoglu et al. 2020). Artificial selection was based on changes in maximal running distance measured during a standardized treadmill endurance test conducted before and after exercise training. To ensure consistency and minimize motivational variability, only rats that consistently completed the exercise protocol were selected for breeding. In the untrained state, both LRT and HRT rats exhibited comparable levels of maximal treadmill running distance and showed no differences in VO_2max_ and cardiorespiratory fitness before training (Koch and Britton 2001; Koch et al. 2013; Wisløff et al. 2015). However, after training, the HRT rats demonstrated significant improvements in running distance and VO_2max_ and other markers of cardiorespiratory fitness, including cardiac muscle cell function. In contrast, LRT rats, despite successfully completing the training, showed minimal changes or even decreases in these same fitness indicators. It is important to note that cardiorespiratory fitness is a strong predictor of all‐cause mortality (Blair et al. 1996; Kokkinos et al. 2008; Kodama 2009). Therefore, the distinctions between HRT and LRT rats only became apparent in response to training (Figure 1), emphasizing the differences in their adaptive responses to exercise rather than inherent baseline fitness levels (Wisløff et al. 2015).

This article provides an overview of a collaborative series of studies using LRT and HRT rat models and the impact of aerobic exercise capacity on neuromuscular adaptations. First, we address skeletal muscle adaptations and the molecular signaling involved, and then we discuss the neuroendocrine‐driven adaptations in rats bred with inherited low and high trainability. Finally, we provide a conclusion regarding the potential signaling pathways involved in the metabolic functioning of neuromuscular adaptations observed in the genetic models, LRT and HRT rats (Table 1).

**TABLE 1:** List of the studies using LRT and HRT rats.

General findings about LRT versus HRT models	References
Aerobic hypothesis posits that *variation in capacity for oxygen metabolism is the central mechanistic determinant of the divide between complex disease and health*	Koch and Britton (2008)
After multiple generations of selective breeding, two lines of rats were developed: HRT and LRT. HRT rats showed significantly greater improvements in running distance following the training program compared to LRT rats	Koch et al. (2013)
HRT rats showed marked increase on VO_2max_ and cardiorespiratory fitness in response to exercise training	Koch et al. (2013), Wisløff et al. (2015)
LRT rats exhibited pronounced metabolic dysfunction, characterized by insulin resistance and increased adiposity, as well as impaired muscle adaptations, characterized by impaired exercise‐induced angiogenesis	Lessard et al. (2013)
HRT rats exhibited better mitochondrial biogenesis and antioxidant capacity compared to LRT rats	Marton et al. (2015)
HRT rats showed greater improvements in muscle mass and strength in response to resistance training compared to LRT rats	Ahtiainen et al. (2018)
LRT rats exhibited more pronounced denervation and cellular senescence in skeletal muscle	Brown, Judge, et al. (2019)
HRT rats retained the ability to improve exercise capacity into late life	Brown, Macpherson, et al. (2019)
SPEG expression correlated with high exercise responsiveness and may be involved in modulating JNK and TGF‐β signaling pathways, which are important in regulating muscle adaptation to exercise	Kusić et al. (2020)
Hyperglycemia modifies the ECM and mechanical signaling in muscle, impairing vascularization and aerobic remodeling, which may also contribute to the low responsiveness observed in LRT animals	MacDonald et al. (2020)
HRT rats exhibit a more pronounced myokine response, particularly in pathways linked to inflammation, metabolism, and muscle remodeling, suggesting a greater capacity for beneficial adaptation to exercise	Farrash et al. (2021)
LRT rats exhibited limited muscle hypertrophy and increased inflammatory and stress‐related genes in response to endurance training	West et al. (2021)
LRT rats experienced greater muscle atrophy, and a more pronounced dysregulation of muscle‐related gene expression compared to HRT rats	Thompson et al. (2022)
HRT rats exhibited greater magnitude of AHN in response to aerobic exercise	Nokia et al. (2016)
HRT rats demonstrated greater improvements in learning and memory, along with enhanced neuroplasticity and cognitive function, following aerobic training	Marton et al. (2016)
HRT rats exhibited better regulation of fear‐associated memories, better stress resilience, improved memory retention, and lower anxiety levels compared to LRT rats	Vanderheyden et al. (2021)

Abbreviations: HRT, high responders to endurance training; LRT, low responders to endurance training; VO_2max_, maximal oxygen consumption.

## 2 Skeletal Muscle Adaptations

**FIGURE 2:**
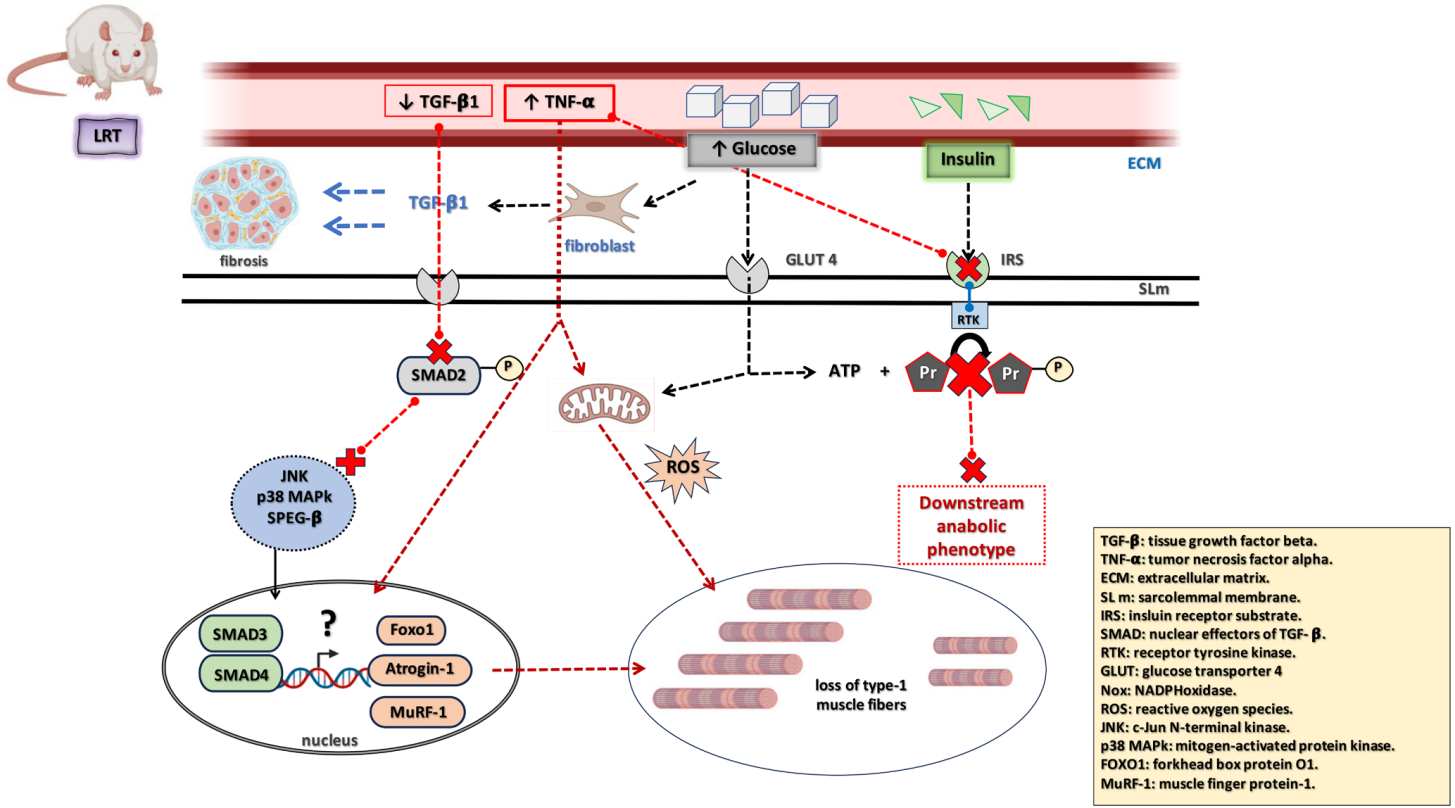
Integrative cellular signaling triggered by impaired glucose tolerance or impaired insulin sensitivity, mitochondrial dysfunction, mediated by intricate cellular signaling orchestrated by TGF‐ß1‐JNK and TNF‐α‐MAPK increased extracellular matrix (ECM) remodeling pathways, and loss of type I muscle fibers in LRT models. *Adapted from* Lessard et al. (2013). TGF‐β1, tissue growth factor beta; TNF‐α, tumor necrosis factor alpha; ECM, extracellular matrix; SL m, sarcolemmal membrane; IRS, insulin receptor substrate; SMAD, nuclear effectors of TGF‐β; RTK, receptor tyrosine kinase; GLUT4, glucose transporter 4; Nox, NADPH oxidase; ROS, reactive oxygen species; JNK, c‐Jun N‐terminal kinase; p38 MAPK, mitogen‐activated protein kinase; SPEG‐β, striated muscle‐specific serine/threonine‐protein kinase beta; FOXO1, forkhead box protein O1; MuRF‐1, muscle finger protein‐1.

Early studies by Lessard et al. (2013) showed dramatic metabolic dysfunction in the LRT model, as evidenced by impaired glucose tolerance, whole‐body insulin sensitivity, and elevated circulating insulin concentration. Elevated body and gonadal fat pad weights, higher plasma triglycerides, and higher circulating leptin were also observed in LRT rats. Moreover, although inflammatory mediators were unaltered before training, LRT rats exhibited higher circulating levels of the inflammatory cytokine tumor necrosis factor (TNF‐α) 48 h post‐training, while the anti‐inflammatory cytokine transforming growth factor β1 (TGF‐ß1) only increased in the HRT group, suggesting that the inflammatory response is not solely regulated by intrinsic aerobic capacity but also by a contribution from the training response phenotype (Lessard et al. 2013). In fact, the TGF‐ß1 cytokine is robustly associated with pathological fibrosis in many organs, including skeletal muscle (Ismaeel et al. 2019). Conversely, markers of mitochondrial density (citrate synthase activity in whole‐muscle lysates) and mitochondrial function (maximal ATP production rates in isolated mitochondria) from oxidative muscle types were similar between LRT and HRT rats in their non‐trained state, and interestingly, LRT showed normal increases in both mitochondrial density and function when submitted to an exercise training regimen. However, the percentage of type I (oxidative) muscle fibers in LRT was significantly lower than HRT rats. Therefore, even without changes in mitochondrial density between the groups, the muscle fiber composition shows pronounced differences between the models and likely contributed to impaired insulin sensitivity in LRT rats. Angiogenesis, in turn, was not different between LRT and HRT rats under untrained conditions; however, it was significantly increased in the skeletal muscle of HRT after exercise training compared to LRT. A diminished blood flow in LRT muscle during exercise training could markedly affect the peripheral oxygen extraction to the abdominal viscera and kidneys. In accordance with the physiological findings, LRT rats not only had lower levels of circulating TGF‐ß1 and higher levels of TNF‐α after exercise training (vs. HRT), but LRT rats also showed hyperphosphorylation of the downstream protein SMAD2 and P38 mitogen‐activated protein kinases, p38 MAPK, along with hyperactivation of c‐Jun N‐terminal kinases (JNKs). This suggests that a complex, cell‐specific interaction between the TGF‐ß‐JNK and TNF‐α‐MAPK pathways (Figure 2) plays a pivotal role in shaping the LRT phenotype—affecting skeletal muscle remodeling, energy transfer, and consequently, impairing the metabolic adaptation of skeletal muscle to exercise (Lessard et al. 2013).

Following these relevant findings from Lessard et al. (2013), Marton et al. (2015) raised the question of whether genetic segregation for trainability would be related to factors associated with mitochondrial adaptation. These authors reported that LRT rats showed inherently higher levels of ROS and lower AMPkα activity and NAD^+^/NADH ratio (vs. HRT control), suggesting impaired redox homeostasis and muscular trainability. In addition, citrate synthase activity was reduced in LRT rats submitted to aerobic training compared to trained HRT rats that showed a significant increase, supporting the hypothesis that mitochondrial mass is reduced in conditions of exercise resistance. Furthermore, the aberrant reduction of mitochondrial transcription factor A (TFAM) levels in trained LRT (vs. HRT) is consistent with compromised mitochondrial biogenesis and unfavorable muscle adaptation to training demands (Popov et al. 2018). A study with a TFAM‐knockout mice model has shown elevated fatigue, progressive skeletal muscle atrophy, and reduced mitochondrial oxidative capacity for energy production (Chatel et al. 2021). Noteworthy, high TFAM levels may be robustly associated with lower AMPk activity observed in trained LRT, supporting the detrimental consequences of exercise resistance in skeletal muscle. Besides, the higher mitochondrial fission 1 protein (Fis1) levels in LRT (Marton et al. 2015) support the idea that the exercise‐resistant phenotype triggers cellular processes related to mitophagy, apoptosis, and mitochondrial division (Ihenacho et al. 2021). The study also reported that the levels of TFAM, peroxisome proliferator‐activated receptor gamma coactivator‐1α (PGC‐1α), nuclear respiratory factor 1 (NRF‐1), and Lon protease were upregulated only in HRT rats submitted to treadmill exercise for 3 months (5 times a week, 30 min of daily session), reinforcing the complex differences between the LRT and HRT rats in untrained or trained conditions as well as the better training responses of HRT models for mitochondrial biogenesis and antioxidant capacity adaptations.

In these early studies, where markers of energy production in skeletal muscle were significantly altered in response to different aerobic capacity, it might be speculated that cardiac muscle likewise would be affected. In this sense, clusters of genes for catenin, integrin, and metalloproteinase‐disintegrin family of proteins were downregulated in LRT hearts compared to HRT, which provide molecular support that angiogenesis, neurogenesis, and tissue development were compromised in rats with lower adaptive exercise capacity (Wisløff et al. 2015). Further, left ventricle (LV) cardiomyocyte cells in HRT rats showed better morphological structure (elongated and narrower cell structure), resulting in reduced calculated LV cell volume. While contractility properties were not different between LRT and HRT rats in the sedentary conditions, LRT rats did not present improvements in cardiac function after high intensity interval training. Instead, LRT rats displayed maladaptive reductions in LV cell length and volume after aerobic training compared to HRT rats. In the same study, osteoglycin (OGN), ranked as the greatest differentially expressed genes (DGEs), was increased in LRT (vs. HRT), indicating pathological extracellular matrix (ECM) remodeling in settings of exercise resistance. OGN is a glycosylated protein in the ECM that regulates the assembly and organization of collagen fibers, acting as a “scaffold” for collagen fibrillogenesis within the matrix (Nulali et al. 2022). OGN can also be cleaved by metalloproteinases (MMPs) during ECM remodeling (Barascuk et al. 2011). Furthermore, OGN has been implicated in TGF‐ß‐mediated cardiac hypertrophy of rats (Jazbutyte et al. 2013; Deckx et al. 2016), pathogenesis of cardiovascular fibrosis (Zuo et al. 2022), and metabolic syndrome (Deckx et al. 2016).

In addition to studies showing better performance and improvements in the muscle adaptation of rats bred for high response to aerobic exercise training, Ahtiainen et al. (2018) explored the hypothesis that HRT rats would have greater skeletal muscle adaptations to a resistance training protocol compared to LRT rats. Rats underwent an 8‐week treadmill exercise training regimen (3 times/week) and maximal running capacity was tested before and after the training period to determine the aerobic training‐related phenotype response. Following the treadmill training, rats were randomly assigned to either resistance training or sedentary control. Here, the focus was on resistance training responses of LRT and HRT models in terms of skeletal muscle adaptations after 6 weeks of ladder‐climbing training. A greater increase in strength (maximal amount of weight carried up during the climb) was observed in HRT rats when compared with LRT. Even though HRT seemed stronger than LRT by carrying up heavier loads along the ladder, morphological differences in muscular adaptations did not accompany the better performance observed in trained HRT. There was also no increase in total body fat mass in HRT rats after the ladder‐climbing resistance training to suggest a potential inverse relationship between muscular strength and fat accumulation. This was further supported by the observation of greater fat accumulation in HRT rats without resistance training, as well as in LRT rats both with and without resistance training. In view of no notable hypertrophy and little changes in physiological adaptation, the authors suggested that the elevated muscular strength observed in HRT rats might be determined by neural‐driven responses rather than muscular adaptations and thus implying that the central nervous system (CNS) is likely overactivated in HRT models.

Several studies highlight the concern that reduced aerobic endurance, coupled with the reduction in skeletal muscle mass, loss of muscle strength, and muscle weakness is a significant issue for the elderly (Goodpaster et al. 2006; Tieland et al. 2018; Wilkinson et al. 2018). In line with clinical evidence exhibiting age‐associated decline in aerobic exercise capacity (Bai et al. 2021), Brown, Judge, et al. (2019) sought to investigate whether the combination of aging and distinct adaptive aerobic capacities would affect key health outcomes, including skeletal muscle health. Aged LRT and HRT female rats (22 months old) were submitted to treadmill running for 16 weeks (2–3 times a week at 60% of their maximum tested running speed). After the training, aged HRT rats showed a marked increase (%) in extensor digitorum longus (EDL) muscle fragmented endplates (vs. aged LRT), which suggests that the muscles of aged HRT rats were stronger than those of aged LRT rats. The increased force production in the HRT rats suggests that they had a better response to aerobic training, which contributed to their enhanced muscle strength. However, when comparing the size of nerve endplates (which help control muscles) between aged and adult rats (11 months old), there were no differences in size for either the LRT or HRT rats. But, as rats aged, their nerve endplates became more fragmented—a sign of aging—regardless of whether they exercised or not (Brown, Judge, et al. 2019). This indicates that while aerobic training can enhance muscle strength, it may not necessarily prevent age‐related structural changes at the neuromuscular junction in either HRT or LRT rats. Further, in the same study, gene expression of denervation associated markers (Myogenin), Acetylcholine Receptor α (AChRα), Muscle‐Specific Kinase (MuSK), Rapsyn, (Rapsyn and GADD45α) were assessed in tibialis anterior (TA) and gastrocnemius (GNT) muscle of rats. While HRT rats displayed lower expression levels of these markers (vs. LRT rats), the aged HRT and LRT rats showed increased expression of denervation markers compared to their younger counterparts. This reflects the typical age‐related decline in neuromuscular function. Interestingly, rats subjected to exercise exhibited lower levels of denervation markers compared to sedentary rats. This suggests that aerobic exercise helps maintain better neuromuscular function by reducing the expression of markers associated with muscle denervation. Likewise, levels of senescence markers (Cdkn2d and Rb1) were downregulated in HRT rats and exercised rats generally had lower levels of senescence markers compared to their sedentary counterparts, indicating better cellular health and adaptation to exercise. This is in accord with other studies that suggest aerobic exercise, such as treadmill running, is capable of reducing the age‐associated accumulation of senescent cells and promoting their clearance (Zhang et al. 2022; Carapeto et al. 2023). These findings suggest a protective effect of maintaining aerobic capacity against aging‐associated skeletal muscle maladaptation.

As the neuromuscular changes were more pronounced in HRT than LRT rats (Brown, Judge, et al. 2019), the researchers further investigated whether aged HRT rats would have better mitochondrial function than LRT rats by measuring markers associated with mitochondrial capacity, quality, and protection from oxidative stress (Brown, Macpherson, et al. 2019). The preservation of exercise capacity in aged HRT rats was related to enhanced mitochondrial biogenesis and quality. This is evident from the elevated protein levels of PGC‐1α (a key regulator of metabolic gene activation, controlling substrate utilization and mitochondrial biogenesis) in TA and TFAM (mitochondrial transcription factor) in gastrocnemius (GTN) muscles of aged HRT rats, indicating robust mitochondrial biogenesis. Additionally, there was an increase in clearance of damaged organelles, indicated by the upregulation of Bcl‐2/adenovirus E1B 19 kDa interacting protein 3 (BNIP3) in TA muscles of aged HRT rats. While PTEN‐induced kinase 1 (PINK1) was reduced in GTN, BNIP3 levels were elevated in TA muscles of aged HRT rats. PINK1 promotes cell survival and protects against mitochondrial dysfunction caused by oxidative stress (Matsuda et al. 2013). In contrast, BNIP3 is involved in numerous cellular signaling pathways, including mitochondrial dysfunction, mitophagy, and cell apoptosis (Gao et al. 2020) as well as sensing oxidative stress (Kubli et al. 2008) The reduction in PINK1 levels might indicate a lesser need for PINK1‐mediated mitophagy, possibly due to better overall mitochondrial health in HRT rats. Whereas the elevated levels of BNIP3 in aged HRT rats suggest enhanced autophagic activity and improved maintenance of mitochondrial health, which likely contribute to the improved muscle function and exercise capacity observed in these rats. Thus, the higher exercise capacity of aged HRT rats is associated with better mitochondrial biogenesis and antioxidant capacity, suggesting that these rats have more efficient systems for maintaining muscle and mitochondrial health, contributing to their enhanced exercise capacity and muscle function compared to LRT rats. Although it is difficult to draw a definite conclusion from a study using three different muscles, the authors pointed out that an increase in mitochondrial volume is not mandatory to improve the exercise capacity of older HRT rats, even considering the lack of changes in mitochondrial proteins of the TA and GTN muscles. Therefore, it is plausible that the improvements in exercise capacity in aged, exercised HRT rats might involve other biological processes (i.e., turnover) or organ systems (i.e., cardiovascular system).

In order to gain mechanistic insight into the regulators of the training response and explore whether disease risk is associated with a lack of adaptation to endurance training, researchers conducted proteome profiling of GTN muscles in HRT and LRT rats. This analysis aimed to further investigate the molecular differences that segregate with high versus low responsiveness to endurance exercise training (Kusić et al. 2020). Among 13 proteins that were more abundant in HRT muscles compared to LRT muscles, striated muscle‐specific serine/threonine‐protein kinase beta (SPEGβ) was the most enriched. This higher abundance is strongly associated with the enhanced responsiveness of HRT rats to endurance training. This study also reported that several proteins, including novel targets involved in c‐Jun NH2‐terminal kinase (JNK) signaling, co‐immunoprecipitated with SPEG‐ß in exercised GTN of HRT, which links with the previously reported differences in TGF‐ß signaling (Lessard et al. 2013). JNK signaling is recognized as a regulator of muscle adaptive response to exercise training (Lessard et al. 2018). These interactions suggest that SPEG‐β is involved in various molecular pathways that might contribute to muscle adaptation and enhanced exercise responsiveness (Figure 2).

Consistent with a role of TGF‐ß signaling on skeletal muscle adaptations of LRT models (Lessard et al. 2013), MacDonald et al. (2020) investigated whether extracellular matrix (ECM) accumulation plays a prominent role in the low improvements in aerobic exercise capacity in LRT rats. The study revealed the skeletal muscle ECM accumulation as a predictor of LRT responses (MacDonald et al. 2020). In addition to exhibiting greater skeletal muscle ECM thickening and increased muscle collagen content, LRT muscle had higher levels of glycation compared to HRT. Notably, the glycation signal was colocalized with the ECM compartment, suggesting that disturbances in glucose homeostasis, even in the absence of diabetes, could lead to remarkable changes to the ECM compartment of skeletal muscle. Thus, the muscles not only fail to undergo aerobic remodeling, but also take on a glycolytic phenotype more congruent with resistance exercise‐induced signaling. Therefore, it might be suggestive that glucose levels could drive metabolic disturbances in LRT models by ECM accumulation, which could lead to negative effects on the aerobic adaptation to exercise training.

Furthermore, Kelahmetoglu et al. (2020) aimed to provide cross‐species transcriptional signatures in skeletal muscle that correlate with inherent aerobic exercise capacity and the response to training, using both human and genetically heterogeneous rat models selected for intrinsic (LCR/HCR) and acquired (LRT/HRT) aerobic exercise capacity. In rats, exercise capacity is associated with circulatory factors such as angiogenesis and tissue oxygenation, as well as genes involved in lipid metabolism and the exercise response associated with ECM remodeling. Low response to training is associated with a unique gene sets for elevated inflammatory signatures, increased metabolic dysfunction, and a potential link to circadian rhythm disruptions. On the other hand, human transcriptome comparison highlighted epigenetic mechanisms linked to exercise capacity and links to damage repair for training response (Kelahmetoglu et al. 2020). Furthermore, increased levels of Foxo1 were observed as a common factor for response signatures in both human and rat. Foxo1 is a transcription factor that regulates the expression of genes involved in muscle atrophy. It induces the expression of atrophy‐related ubiquitin ligases, such as atrogin‐1 and MuRF1, which are crucial for protein degradation in muscle cells (Sandri et al. 2004; Xu et al. 2012). The presence of higher Foxo1 levels in LRT indicates that these rats might experience more muscle stress and damage, requiring increased protein turnover and degradation. This could be a factor contributing to their lower adaptation to exercise training. The study also highlights the role of angiogenesis in enhancing aerobic capacity, particularly in high responders, and suggests that differences in angiogenic potential may underlie the varying responses to exercise training (Rahimi et al. 2022).

To gain further understanding of the molecular interaction in skeletal muscle of LRT and HRT models, upstream drivers of exercise response were investigated by Farrash et al. (2021). The authors examined a collection of myokines in LRT and HRT models, supported by the hypothesis that greater myokine concentrations would be observed in HRT rats (vs. LRT) at baseline and in response to acute exercise training (Farrash et al. 2021). This distinction in myokine profiles was expected to reflect different health and adaptive traits between the two groups, assuming that myokines underlie some of the health benefits of exercise. Six myokines were appropriately detectable in the plasma: Irisin, Brain‐derived neurotrophic factor (BDNF), Secreted Protein Acidic and Rich in Cysteine (SPARC), Fractalkine, Fibroblast growth factor 21 (FGF21), and Musclin. FGF21 and Musclin were elevated in LRT rats (vs. HRT) at baseline condition. Three weeks of daily exercise training increased Musclin levels in HRT rats and decreased FGF21 levels in LRT rats. FGF21 is involved in regulating mitochondrial function. Dysregulation of these myokines can lead to long‐term impairments in muscle energy metabolism (Piccirillo 2019). In response to acute training, SPARC and Irisin levels were increased in both LRT and HRT traits, while BDNF concentration reduced in both models post‐training. Moreover, Fractalkine was increased only in HRT (vs. LRT), which was an unexpected finding. The study concluded that exercise training modifies myokine responses to acute exercise, but these responses were not robustly linked to adaptive potential in aerobic capacity. This suggests that myokines may not be the primary regulators of adaptive traits related to exercise capacity. However, it is important to note that LRT‐related high baseline levels in both FGF2 and Musclin may indicate a protective role of myokines against metabolic stress and cardiac protection (Szaroszyk et al. 2022; Harris et al. 2023). Moreover, some myokines regulate muscle vascularization. Long‐term alterations in their secretion can affect blood supply to muscles, impacting nutrient delivery and waste removal (Chen et al. 2024).

Following the fact that the LRT model has poor metabolism and angiogenesis in addition to dysregulated molecular signal transduction (Lessard et al. 2013; MacDonald et al. 2020), West et al. (2021) hypothesized that LRT would exhibit attenuated hypertrophy compared with HRT in line with distinct enrichments for gene sets of biological processes (West et al. 2021). To create a functional overload and induce compensatory hypertrophy of the plantaris muscle, LRT and HRT models underwent unilateral ablation of the gastrocnemius (GTN) and soleus (SOL) muscles. In response to functional overload, LRT rats had significantly attenuated hypertrophy compared to HRT rats (20.1% vs. 41.6% increase). Further, 96 upregulated and 101 downregulated pathways differed in magnitude between LRT and HRT, whereas 27 pathways showed opposite regulation (up vs. down) in LRT compared to HRT, suggesting differences in protein metabolism and cellular processes. This suggests that the genetic factors influencing endurance training adaptation may also affect hypertrophic responses to resistance training. Importantly, the study provides evidence that poor aerobic adaptability may be associated with dysregulation of transcriptional processes involved in adaptation to mechanical overload. It is also likely that the genetic factors underlying poor aerobic adaptation may also dysregulate transcriptional processes involved in adaptation to mechanical overload, which could indirectly affect post‐translational events. LRT rats showed impaired gene pathways/programs for post‐translational protein assembly and transport in association with anabolic resistance, which indicates that rats with low response for endurance training had impaired skeletal muscle hypertrophy in response to functional overload. The two biological processes downregulated in LRT and upregulated in HRT were categorized in “Response to endoplasmic reticulum stress” and “Golgi vesicle transport/organization and localization”. Specifically, the unfolded protein response (UPR) pathway, which is activated in response to ER stress, showed different regulation patterns between the two groups. This suggests that LRT rats may have an impaired ability to handle protein folding stress during muscle growth compared to HRT rats. Likewise, genes involved in Golgi organization and vesicle trafficking showed altered expression patterns in LRT rats compared to HRT rats during muscle hypertrophy. This indicates that the genetic factors influencing poor aerobic adaptation in LRT rats may also affect intracellular protein transport processes, potentially contributing to their reduced hypertrophic response. The implications for those responses rely on the disrupted synthesis, folding, and structural integrity of cellular proteins (Afroze and Kumar 2019). Therefore, the genetic differences between HRT and LRT rats not only affect their response to endurance training but also influence cellular processes related to protein folding, ER stress response, and intracellular protein transport during muscle hypertrophy. The dysregulation of these pathways in LRT rats may contribute to their attenuated muscle growth response compared to HRT rats.

Seeing that poor metabolism and dysregulated stress/inflammatory and TGFß‐associated signaling were observed in LRT (Lessard et al. 2013), Thompson et al. (2022) hypothesized that the genetic factors that influence aerobic training adaptation also affect muscle fiber plasticity, and therefore, the low responders to endurance training (LRT) model would exhibit aberrant muscle disturbances compared to high responders to endurance training (HRT) when subjected to muscle immobilization (Thompson et al. 2022). These investigators previously reported changes in gene transcription profiles of LRT and HRT in response to functional overload, and both common and distinct biological process enrichment were apparent in LRT and HRT models for compensatory hypertrophy (see above) (West et al. 2021). As a follow‐up study, aged female rats (14 months of age) were submitted to hindlimb immobilization via casting for 3 days to induce atrophy of the plantaris and soleus muscles. The study showed that aged LRT had exacerbated atrophy compared to HRT following immobilization, suggesting a link between poor endurance training response and increased susceptibility to disuse atrophy with aging. The muscle atrophy was more pronounced in type I muscle fiber type (slow‐twitch, high oxidative capacity) than type II (fast‐twitch low oxidative capacity) because plantaris (predominantly comprised of type II muscle fibers) atrophy occurred in smaller degree between LRT and HRT rats, while the resulting gene‐set enrichments were similarly up/down regulated but different in the magnitude of response.

**FIGURE 3:**
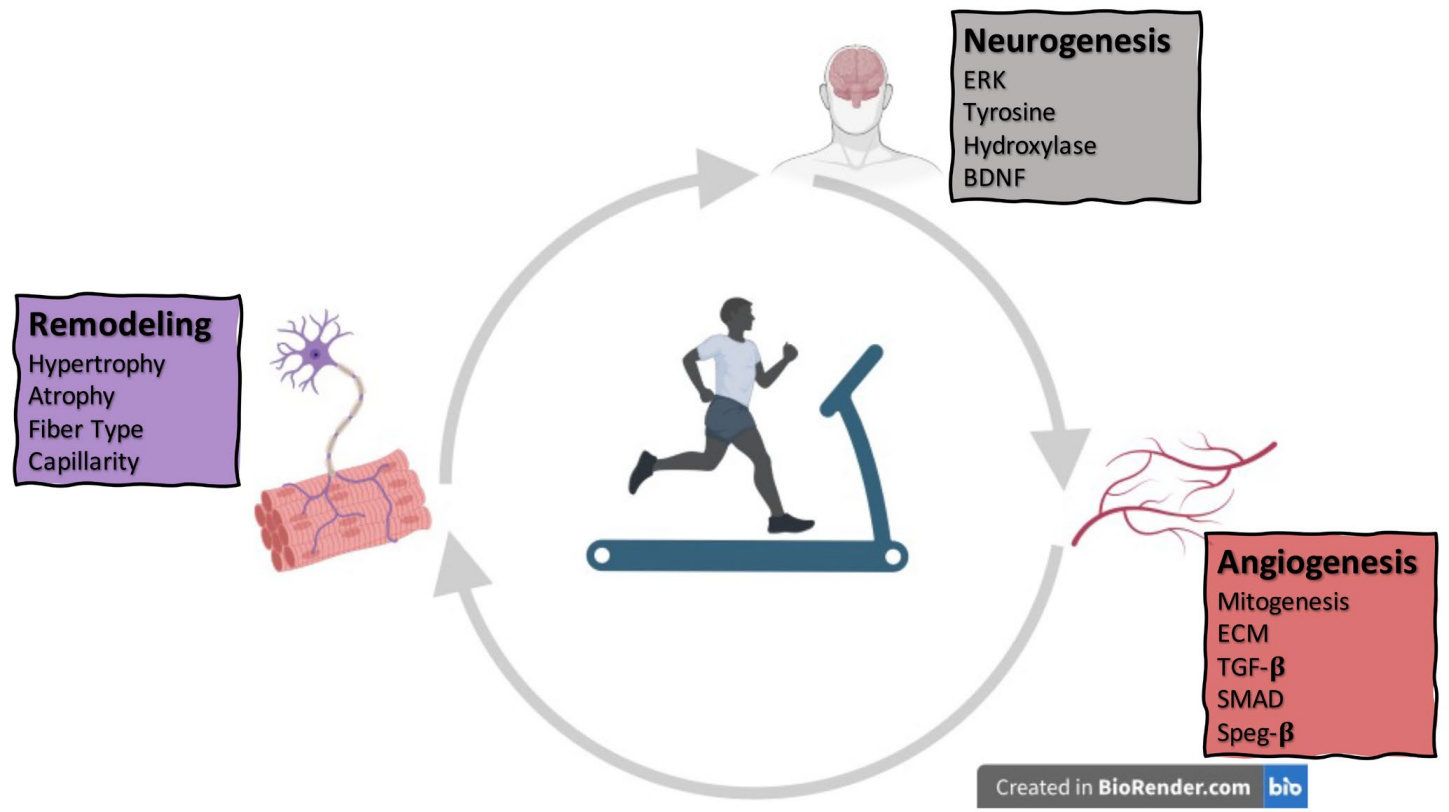
Neurogenesis response, muscle remodeling, and the excitability of the central nervous system are improved in a rat model of high response to training (HRT). Neurogenesis is mediated by increased levels of brain‐derived neurotrophic factor (BDNF) (Marton et al. 2016), greater activation of extracellular signal‐regulated kinase (ERK) in the hippocampus and amygdala, and reduced tyrosine hydroxylase levels in the locus coeruleus (Vanderheyden et al. 2021). Angiogenesis associates with mitogenesis, TGF‐β‐JNK/SMAD signal transduction, and high levels of serine/threonine‐protein kinase beta (SPEGβ) in skeletal muscle (Lessard et al. 2013, 2018). Enhanced skeletal muscle remodeling, including the balance between the hypertrophic and atrophic processes, fiber type, and capillarity, is observed in HRT rats. Genetic traits associated with better endurance training adaptations may also confer advantages in preserving muscle mass during periods of inactivity and immobility, especially in slow‐twitch muscle fibers (MacDonald et al. 2020; West et al. 2021). HRT‐related responses may reflect an inherent protective mechanism to stress events. TGF‐β, tissue growth factor beta. JNK, c‐Jun N‐terminal kinase. SMAD, nuclear effectors of TGF‐β.

Further, LRT showed a significant decrease in muscle protein synthesis (in soleus muscle) after immobilization. Rapid atrophy in the early response to immobilization in soleus of LRT compared to HRT was associated with divergent enrichment of numerous biological processes, such as autophagy. Differences in the magnitude of negative enrichment in calcium ion transport were also evident between LRT and HRT. In addition, there was divergent enrichment for carbohydrate metabolism processes in HRT and LRT plantaris muscle; LRT showed reduced capacity to maintain cell processes for muscle glucose metabolism during disuse. In fact, stiffer ECM and changes in its composition can promote a more glycolytic metabolic state in the cells (Ge et al. 2021). This metabolic impairment may contribute to decreased protein synthesis. The largest shift in gene‐set enrichments between HRT and LRT in soleus muscle was for immune, cytokine regulation, and lymphocyte activation biological processes. In plantaris muscle, the largest biological process shift was a downregulation of numerous nucleotide metabolism pathways between LRT and HRT rats compared to their respective control groups (Thompson et al. 2022). Since nucleotide synthesis is required for RNA production and new protein synthesis during adaptation, the downregulation of nucleotide metabolism pathways may contribute to impaired transcription/translation programs in LRT (Lane and Fan 2015). HRT soleus muscle, in turn, showed resistance to the muscle atrophy after immobilization and maintained muscle mass comparable to HRT control animals not subjected to immobilization. This observed resistance to muscle loss can be attributed to inherited genetic factors resulting from selective breeding for enhanced endurance training response. These genetic factors appear to offer protection against short‐term muscle disuse, particularly in type I muscle fibers, which are predominant in the soleus muscle. This suggests that the genetic traits associated with better endurance training adaptations may also confer advantages in preserving muscle mass during periods of inactivity, especially in slow‐twitch muscle fibers. Therefore, it is very likely that there is a correlation between genetic elements that govern endurance training adaptations and the susceptibility to muscle atrophy during periods of disuse, suggesting that the same genetic factors that make someone a “low responder” to endurance training might also predispose them to more rapid muscle loss during periods of disuse or immobilization (Figure 3).

## 3 Neural‐Driven Adaptations in Rats Bred With Inherited Low and High Trainability

There are accumulating evidences indicating that physical exercise enhances hippocampal and cognitive functions in rodents as well as in humans, and these exercise‐induced improvements may help slow down the cognitive decline typically associated with aging, and potentially reduce the risk of developing neurodegenerative diseases (Marlatt et al. 2012; Tharmaratnam et al. 2017; Lei et al. 2019). Nokia et al. (2016) conducted a study on rats that provided strong evidence for the link between aerobic exercise and adult hippocampal neurogenesis (AHN). Their findings revealed evidence that neurogenesis in the hippocampus is greater in HRT rats compared to LRT rats, and especially when the aerobic exercise involves long duration (Nokia et al. 2016). The authors hypothesized that genetic predisposition would not play a prominent role in AHN under untrained conditions but would promote greater AHN in exercised HRT rats than in LRT rats. This study tested three different exercise training protocols: rats subjected to treadmill training, voluntary wheel running fitted in their cages, and resistance ladder climb training. AHN amount was significantly increased in both LRT and HRT rats submitted to aerobic exercise (voluntary wheel running or treadmill training) in comparison to those that performed resistance training (ladder climb training). As a better aerobic training performance was observed in HRT (running distance and speed) than in LRT rats, the amount of AHN was also increased in HRT rats compared with LRT rats. This suggests that the duration and sustained nature of exercise are crucial factors in promoting AHN, rather than just intensity alone. Noteworthy, physical exercise most effectively promotes adult hippocampal neurogenesis when it is aerobic and sustained, especially in individuals with a genetic predisposition to respond well to exercise.

The skeletal muscle adaptation to endurance training involves both peripheral and central mechanisms, including significant contributions from altered neural input. Neural adaptations influence muscle performance by modulating motor unit recruitment patterns, firing rates, and synaptic efficiency within spinal and supraspinal circuits (Mettler and Griffin 2016; Jones et al. 2023). Moreover, endurance training can induce plastic changes in spinal cord circuitry, including increased excitability of motoneurons and changes in presynaptic inhibition, which collectively improve neuromuscular transmission and coordination (Gardiner 2006; Johnson et al. 2017). Altered afferent feedback from muscle proprioceptors also contributes to the fine‐tuning of motor output and may facilitate more efficient locomotor patterns (Rossignol et al. 2006; Molkov et al. 2024). Therefore, the adaptive response of skeletal muscle to endurance stimuli is not solely dependent on intrinsic muscle properties but is also shaped by neural adaptations that refine motor control and performance. This emphasizes the importance of considering neuromuscular integration when interpreting training‐induced muscle adaptations in experimental models. These adaptations may explain part of the improved functional capacity observed in HRT rats and support the idea that muscle performance gains are not only strictly due to intrinsic changes in fiber composition or mitochondrial density, but also to reorganization within the neural circuits controlling locomotion (Roy et al. 1991; de Leon et al. 1994).

Accordingly, Marton et al. (2016) hypothesized that genetic segregation for trainability could affect brain function and central signaling processes (Marton et al. 2016). The authors reported greater improvements in aerobic capacity in HRT rats compared to LRT rats after training. Brain‐derived neurotrophic factor (BDNF) level, which is crucial for neuronal growth and survival (Lipsky and Marini 2007), increased more significantly in HRT rats than in LRT rats following exercise. Moreover, HRT rats demonstrated better performance in cognitive tasks, particularly those related to learning and memory, compared to LRT rats. Exercise training reduced oxidative damage in the brain, with a more pronounced effect in HRT rats. This suggests that the rate of training response affects the brain's ability to manage oxidative stress. Based on these findings that revealed greater improvements in brain function, including enhanced neuroplasticity and cognitive performance in HRT rats, the degree of improvement in aerobic capacity from exercise training is not localized to skeletal muscles, but is also associated with the extent of cognitive benefits, emphasizing the link between cardiovascular fitness and brain health.

Considering the findings concerning the direct role of aerobic exercise capacity on brain function (Nokia et al. 2016; Marton et al. 2016), Vanderheyden et al. (2021) sought to investigate whether distinct sensitivity to aerobic training would affect stress‐related neurobiological changes and regulate fear‐associated memory processing. They hypothesized that inherited variation for aerobic gain in response to physical training might also impact the brain's adaptability in areas related to stress, memory formation, and anxiety pathways. Their study demonstrated that LRT and HRT rats exhibited different behavioral responses to acute stress. HRT rats showed impaired fear‐associated memory processing following fear conditioning and exhibited a lack of extinction and recall, suggesting diminished processing of fear‐associated memories. HRT rats also had elevated adrenocorticotropic hormone (ACTH) levels, indicating a heightened stress response (Knezevic et al. 2023); greater phospho extracellular signal‐regulated kinase (ERK) activation in the hippocampus and amygdala, as well as reduced tyrosine hydroxylase levels in the locus coeruleus, suggesting a loss of noradrenergic cells and may suggest that the behavioral impairments were due, at least in part, to either molecular signaling or structural abnormalities in the noradrenergic signaling (Vanderheyden et al. 2021). The ERK activation was suggested as a unique molecular pathway underlying stress neural adaptations and high aerobic capacity. These findings provide valuable insights into the complex relationship between exercise adaptability, stress response, and fear‐associated memory processing. They suggest that the genetic factors influencing exercise responsiveness may have broader implications on an individual's neurological plasticity and behavioral responses regulated by stress.

## 4 Conclusion

Physical activity plays a crucial role in mitigating the negative effects of sedentary behavior on metabolism and improving various health outcomes. Aerobic exercise is particularly effective in addressing numerous clinical conditions, while resistance training enhances strength and muscular mass. Aerobic capacity can be considered a trait resulting from the interplay between intrinsic and adaptive genes and the environment. The development of genetically heterogeneous rat models for low and high aerobic performance on a speed‐ramped treadmill running tests (Koch et al. 2012; Koch and Britton 2019) offers a valuable preclinical experimental framework. This approach not only illuminates the mechanistic links between low exercise capacity and increased disease susceptibility but also scrutinizes the protective effects associated with high aerobic capacity.

**FIGURE 4:**
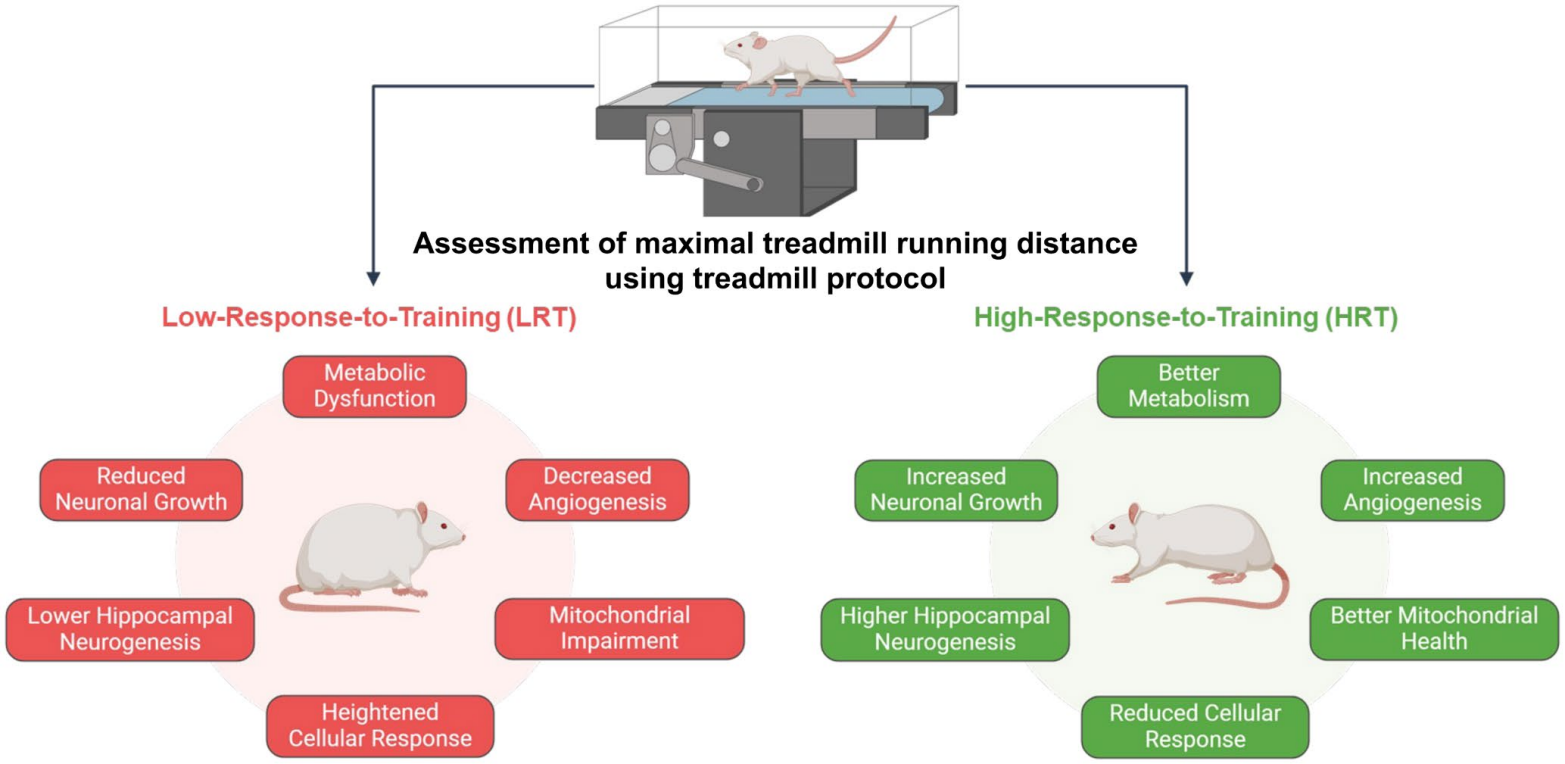
A representation of the major responses observed in rats bred for low (red color) and high (green color) response to aerobic exercise training.

Therefore, this review has summarized the intricate molecular and cellular mechanisms underlying skeletal muscle adaptations in the LRT rat model with poor adaptive response to exercise in comparison to the HRT rat model, which responds positively to the endurance training (Figure 4). The LRT‐related adverse effects in neuromuscular responses seem to be primarily driven by: (i) impaired glucose tolerance or impaired insulin sensitivity, (ii) increased ECM remodeling, (iii) loss of type I muscle fibers, (iv) mitochondrial dysfunction, and (v) an intricate cellular signaling orchestrated by TGF‐ß1‐JNK and TNF‐α‐MAPK pathways. On the other hand, the HRT rat model demonstrates increased central nervous system excitability, which might reflect a protective mechanism to stress events.

These findings highlight the multifaceted nature of exercise capacity and its extensive implications for health and disease. Further research using the theory‐based selectively bred rat exercise models for intrinsic and adaptive exercise capacity (LCR/HCR and LRT/HRT) promises to yield valuable insights for both preventive and therapeutic interventions targeting exercise‐related physiological adaptations.

## Conflicts of Interest

The authors declare no conflicts of interest.

## Data Availability

The data that support the findings of this study are available from the corresponding author upon reasonable request.

## References

[cph470029-bib-0001] Afroze, D. , and A. Kumar . 2019. “ER Stress in Skeletal Muscle Remodeling and Myopathies.” FEBS Journal 286: 379–398. 29239106 10.1111/febs.14358 PMC6002870

[cph470029-bib-0002] Ahtiainen, J. P. , S. Lensu , I. Ruotsalainen , et al. 2018. “Physiological Adaptations to Resistance Training in Rats Selectively Bred for Low and High Response to Aerobic Exercise Training.” Experimental Physiology 103: 1513–1523. 30184287 10.1113/EP087144 PMC6519084

[cph470029-bib-0003] Bai, X. , K. G. Soh , R. D. Omar Dev , et al. 2021. “Aerobic Exercise Combination Intervention to Improve Physical Performance Among the Elderly: A Systematic Review.” Frontiers in Physiology 12: 798068. 35058805 10.3389/fphys.2021.798068 PMC8764279

[cph470029-bib-0004] Barascuk, N. , E. Vassiliadis , Q. Zheng , et al. 2011. “Levels of Circulating MMCN‐151, a Degradation Product of Mimecan, Reflect Pathological Extracellular Matrix Remodeling in Apolipoprotein E Knockout Mice.” Biomarker Insights 6: 7777. 10.4137/BMI.S7777 PMC3201086 22084568

[cph470029-bib-0005] Blair, S. N. , J. B. Kampert , H. W. Kohl , et al. 1996. “Influences of Cardiorespiratory Fitness and Other Precursors on Cardiovascular Disease and All‐Cause Mortality in Men and Women.” JAMA 276: 205–210. 8667564

[cph470029-bib-0006] Bouchard, C. , P. An , T. Rice , et al. 1999. “Familial Aggregation of VO(2max) Response to Exercise Training: Results From the HERITAGE Family Study.” Journal of Applied Physiology (1985) 87: 1003–1008. 10.1152/jappl.1999.87.3.1003 10484570

[cph470029-bib-0007] Bouchard, C. , and T. Rankinen . 2001. “Individual Differences in Response to Regular Physical Activity.” Medicine and Science in Sports and Exercise 33: S446–S451. 11427769 10.1097/00005768-200106001-00013

[cph470029-bib-0008] Brown, L. A. , J. L. Judge , P. C. Macpherson , et al. 2019. “Denervation and Senescence Markers Data From Old Rats With Intrinsic Differences in Responsiveness to Aerobic Training.” Data in Brief 27: 104570. 31687430 10.1016/j.dib.2019.104570 PMC6820082

[cph470029-bib-0009] Brown, L. A. , P. C. Macpherson , L. G. Koch , N. R. Qi , S. L. Britton , and S. V. Brooks . 2019. “Late Life Maintenance and Enhancement of Functional Exercise Capacity in Low and High Responding Rats After Low Intensity Treadmill Training.” Experimental Gerontology 125: 110657. 31306740 10.1016/j.exger.2019.110657 PMC6707857

[cph470029-bib-0010] Carapeto, P. , J. Kahng , A. B. T. Alves Wagner , et al. 2023. “374‐OR: Exercise Training Decreases ß‐Cell Senescence in Mice and Humans.” Diabetes 72: 374‐OR. 10.2337/db23-374-OR.

[cph470029-bib-0011] Chatel, B. , S. Ducreux , Z. Harhous , et al. 2021. “Impaired Aerobic Capacity and Premature Fatigue Preceding Muscle Weakness in the Skeletal Muscle Tfam‐ Knockout Mouse Model.” Disease Models & Mechanisms 14: dmm048981. 10.1242/dmm.048981. 34378772 PMC8461820

[cph470029-bib-0012] Chen, Z.‐T. , Z.‐X. Weng , J. D. Lin , and Z.‐X. Meng . 2024. “Myokines: Metabolic Regulation in Obesity and Type 2 Diabetes.” Life Metabolism 3: loae006. 10.1093/lifemeta/loae006. 39872377 PMC11749576

[cph470029-bib-0013] de Leon, R. , J. A. Hodgson , R. R. Roy , and V. R. Edgerton . 1994. “Extensor‐ and Flexor‐Like Modulation Within Motor Pools of the Rat Hindlimb During Treadmill Locomotion and Swimming.” Brain Research 654: 241–250. 7987674 10.1016/0006-8993(94)90485-5

[cph470029-bib-0014] Deckx, S. , S. Heymans , and A.‐P. Papageorgiou . 2016. “The Diverse Functions of Osteoglycin: A Deceitful Dwarf, or a Master Regulator of Disease?” FASEB Journal 30: 2651–2661. 27080639 10.1096/fj.201500096R

[cph470029-bib-0015] Farrash, W. F. , B. E. Phillips , S. L. Britton , et al. 2021. “Myokine Responses to Exercise in a Rat Model of Low/High Adaptive Potential.” Frontiers in Endocrinology 12: 645881. 34177798 10.3389/fendo.2021.645881 PMC8220071

[cph470029-bib-0016] Gao, A. , J. Jiang , F. Xie , and L. Chen . 2020. “Bnip3 in Mitophagy: Novel Insights and Potential Therapeutic Target for Diseases of Secondary Mitochondrial Dysfunction.” Clinica Chimica Acta 506: 72–83. 10.1016/j.cca.2020.02.024 32092316

[cph470029-bib-0017] Gardiner, P. F. 2006. “Changes in ??‐Motoneuron Properties With Altered Physical Activity Levels.” Exercise and Sport Sciences Reviews 34: 54–58. 16672801 10.1249/00003677-200604000-00003

[cph470029-bib-0018] Ge, H. , M. Tian , Q. Pei , F. Tan , and H. Pei . 2021. “Extracellular Matrix Stiffness: New Areas Affecting Cell Metabolism.” Frontiers in Oncology 11: 631991. 33718214 10.3389/fonc.2021.631991 PMC7943852

[cph470029-bib-0019] Giles, L. V. , M. S. Koehle , B. E. Saelens , H. Sbihi , and C. Carlsten . 2021. “When Physical Activity Meets the Physical Environment: Precision Health Insights From the Intersection.” Environmental Health and Preventive Medicine 26: 68. 34193051 10.1186/s12199-021-00990-w PMC8247190

[cph470029-bib-0020] Goodpaster, B. H. , S. W. Park , T. B. Harris , et al. 2006. “The Loss of Skeletal Muscle Strength, Mass, and Quality in Older Adults: The Health, Aging and Body Composition Study.” Journals of Gerontology. Series A, Biological Sciences and Medical Sciences 61: 1059–1064. 17077199 10.1093/gerona/61.10.1059

[cph470029-bib-0021] Harris, M. P. , S. Zeng , Z. Zhu , et al. 2023. “Myokine Musclin Is Critical for Exercise‐Induced Cardiac Conditioning.” International Journal of Molecular Sciences 24: 6525. 37047496 10.3390/ijms24076525 PMC10095193

[cph470029-bib-0022] Ihenacho, U. K. , K. A. Meacham , M. C. Harwig , M. E. Widlansky , and R. B. Hill . 2021. “Mitochondrial Fission Protein 1: Emerging Roles in Organellar Form and Function in Health and Disease.” Frontiers in Endocrinology 12: 660095. 33841340 10.3389/fendo.2021.660095 PMC8027123

[cph470029-bib-0023] Ismaeel, A. , J.‐S. Kim , J. S. Kirk , R. S. Smith , W. T. Bohannon , and P. Koutakis . 2019. “Role of Transforming Growth Factor‐β in Skeletal Muscle Fibrosis: A Review.” International Journal of Molecular Sciences 20: 2446. 31108916 10.3390/ijms20102446 PMC6566291

[cph470029-bib-0024] Jazbutyte, V. , J. Fiedler , S. Kneitz , et al. 2013. “MicroRNA‐22 Increases Senescence and Activates Cardiac Fibroblasts in the Aging Heart.” Age (Dordrecht, Netherlands) 35: 747–762. 22538858 10.1007/s11357-012-9407-9 PMC3636396

[cph470029-bib-0025] Johnson, M. D. , C. K. Thompson , V. M. Tysseling , R. K. Powers , and C. J. Heckman . 2017. “The Potential for Understanding the Synaptic Organization of Human Motor Commands via the Firing Patterns of Motoneurons.” Journal of Neurophysiology 118: 520–531. 28356467 10.1152/jn.00018.2017 PMC5511870

[cph470029-bib-0026] Jones, E. J. , Y. Guo , E. Martinez‐Valdes , et al. 2023. “Acute Adaptation of Central and Peripheral Motor Unit Features to Exercise‐Induced Fatigue Differs With Concentric and Eccentric Loading.” Experimental Physiology 108: 827–837. 37018481 10.1113/EP091058 PMC10988466

[cph470029-bib-0027] Kelahmetoglu, Y. , P. R. Jannig , I. Cervenka , et al. 2020. “Comparative Analysis of Skeletal Muscle Transcriptional Signatures Associated With Aerobic Exercise Capacity or Response to Training in Humans and Rats.” Frontiers in Endocrinology 11: 591476. 33193103 10.3389/fendo.2020.591476 PMC7649134

[cph470029-bib-0028] Kivelä, R. , M. Silvennoinen , M. Lehti , et al. 2010. “Gene Expression Centroids That Link With Low Intrinsic Aerobic Exercise Capacity and Complex Disease Risk.” FASEB Journal 24: 4565–4574. 20643908 10.1096/fj.10-157313 PMC2974413

[cph470029-bib-0029] Knezevic, E. , K. Nenic , V. Milanovic , and N. N. Knezevic . 2023. “The Role of Cortisol in Chronic Stress, Neurodegenerative Diseases, and Psychological Disorders.” Cells 12: 2726. 10.3390/cells12232726. 38067154 PMC10706127

[cph470029-bib-0030] Koch, L. G. , and S. L. Britton . 2001. “Artificial Selection for Intrinsic Aerobic Endurance Running Capacity in Rats.” Physiological Genomics 5: 45–52. 11161005 10.1152/physiolgenomics.2001.5.1.45

[cph470029-bib-0031] Koch, L. G. , and S. L. Britton . 2008. “Aerobic Metabolism Underlies Complexity and Capacity.” Journal of Physiology 586: 83–95. 17947307 10.1113/jphysiol.2007.144709 PMC2375572

[cph470029-bib-0032] Koch, L. G. , and S. L. Britton . 2018. “Theoretical and Biological Evaluation of the Link Between Low Exercise Capacity and Disease Risk.” Cold Spring Harbor Perspectives in Medicine 8: a029868. 28389512 10.1101/cshperspect.a029868 PMC5749140

[cph470029-bib-0033] Koch, L. G. , and S. L. Britton . 2019. “Rat Models of Exercise for the Study of Complex Disease.” 309–317. http://link.springer.com/10.1007/978‐1‐4939‐9581‐3_15. 10.1007/978-1-4939-9581-3_15 31228164

[cph470029-bib-0034] Koch, L. G. , S. L. Britton , and U. Wisløff . 2012. “A Rat Model System to Study Complex Disease Risks, Fitness, Aging, and Longevity.” Trends in Cardiovascular Medicine 22: 29–34. 22867966 10.1016/j.tcm.2012.06.007 PMC3440495

[cph470029-bib-0035] Koch, L. G. , O. J. Kemi , N. Qi , et al. 2011. “Intrinsic Aerobic Capacity Sets a Divide for Aging and Longevity.” Circulation Research 109: 1162–1172. 21921265 10.1161/CIRCRESAHA.111.253807 PMC3236084

[cph470029-bib-0036] Koch, L. G. , G. E. Pollott , and S. L. Britton . 2013. “Selectively Bred Rat Model System for Low and High Response to Exercise Training.” Physiological Genomics 45: 606–614. 23715262 10.1152/physiolgenomics.00021.2013 PMC3727016

[cph470029-bib-0037] Kodama, S. 2009. “Cardiorespiratory Fitness as a Quantitative Predictor of All‐Cause Mortality and Cardiovascular Events in Healthy Men and Women.” JAMA 301: 2024. 19454641 10.1001/jama.2009.681

[cph470029-bib-0038] Kokkinos, P. , J. Myers , J. P. Kokkinos , et al. 2008. “Exercise Capacity and Mortality in Black and White Men.” Circulation 117: 614–622. 18212278 10.1161/CIRCULATIONAHA.107.734764

[cph470029-bib-0039] Kubli, D. A. , M. N. Quinsay , C. Huang , Y. Lee , and A. B. Gustafsson . 2008. “Bnip3 Functions as a Mitochondrial Sensor of Oxidative Stress During Myocardial Ischemia and Reperfusion.” American Journal of Physiology Heart and Circulatory Physiology 295: H2025–H2031. 18790835 10.1152/ajpheart.00552.2008 PMC2614576

[cph470029-bib-0040] Kusić, D. , J. Connolly , H. Kainulainen , et al. 2020. “Striated Muscle‐Specific Serine/Threonine‐Protein Kinase Beta Segregates With High Versus Low Responsiveness to Endurance Exercise Training.” Physiological Genomics 52: 35–46. 31790338 10.1152/physiolgenomics.00103.2019 PMC6985788

[cph470029-bib-0041] Lane, A. N. , and T. W.‐M. Fan . 2015. “Regulation of Mammalian Nucleotide Metabolism and Biosynthesis.” Nucleic Acids Research 43: 2466–2485. 25628363 10.1093/nar/gkv047 PMC4344498

[cph470029-bib-0042] Lei, X. , Y. Wu , M. Xu , O. D. Jones , J. Ma , and X. Xu . 2019. “Physical Exercise: Bulking Up Neurogenesis in Human Adults.” Cell & Bioscience 9: 74. 31508196 10.1186/s13578-019-0337-4 PMC6724373

[cph470029-bib-0043] Lessard, S. J. , T. L. MacDonald , P. Pathak , et al. 2018. “JNK Regulates Muscle Remodeling via Myostatin/SMAD Inhibition.” Nature Communications 9: 3030. 10.1038/s41467-018-05439-3 PMC6072737 30072727

[cph470029-bib-0044] Lessard, S. J. , D. A. Rivas , A. B. Alves‐Wagner , et al. 2013. “Resistance to Aerobic Exercise Training Causes Metabolic Dysfunction and Reveals Novel Exercise‐Regulated Signaling Networks.” Diabetes 62: 2717–2727. 23610057 10.2337/db13-0062 PMC3717870

[cph470029-bib-0045] Lipsky, R. H. , and A. M. Marini . 2007. “Brain‐Derived Neurotrophic Factor in Neuronal Survival and Behavior‐Related Plasticity.” Annals of the New York Academy of Sciences 1122: 130–143. 18077569 10.1196/annals.1403.009

[cph470029-bib-0046] Lujan, H. L. , S. L. Britton , L. G. Koch , and S. E. DiCarlo . 2006. “Reduced Susceptibility to Ventricular Tachyarrhythmias in Rats Selectively Bred for High Aerobic Capacity.” American Journal of Physiology. Heart and Circulatory Physiology 291: H2933–H2941. 16891405 10.1152/ajpheart.00514.2006

[cph470029-bib-0047] MacDonald, T. L. , P. Pattamaprapanont , P. Pathak , et al. 2020. “Hyperglycaemia Is Associated With Impaired Muscle Signalling and Aerobic Adaptation to Exercise.” Nature Metabolism 2: 902–917. 10.1038/s42255-020-0240-7 PMC8278496 32694831

[cph470029-bib-0048] Mandsager, K. , S. Harb , P. Cremer , D. Phelan , S. E. Nissen , and W. Jaber . 2018. “Association of Cardiorespiratory Fitness With Long‐Term Mortality Among Adults Undergoing Exercise Treadmill Testing.” JAMA Network Open 1: e183605. 30646252 10.1001/jamanetworkopen.2018.3605 PMC6324439

[cph470029-bib-0049] Marlatt, M. W. , M. C. Potter , P. J. Lucassen , and H. van Praag . 2012. “Running Throughout Middle‐Age Improves Memory Function, Hippocampal Neurogenesis, and BDNF Levels in Female C57BL/6J Mice.” Developmental Neurobiology 72: 943–952. 22252978 10.1002/dneu.22009 PMC3485396

[cph470029-bib-0050] Marton, O. , E. Koltai , M. Takeda , et al. 2015. “Mitochondrial Biogenesis‐Associated Factors Underlie the Magnitude of Response to Aerobic Endurance Training in Rats.” Pflügers Archiv‐European Journal of Physiology 467: 779–788. 24943897 10.1007/s00424-014-1554-7 PMC4272336

[cph470029-bib-0051] Marton, O. , E. Koltai , M. Takeda , et al. 2016. “The Rate of Training Response to Aerobic Exercise Affects Brain Function of Rats.” Neurochemistry International 99: 16–23. 27262284 10.1016/j.neuint.2016.05.012

[cph470029-bib-0052] Matsuda, S. , Y. Kitagishi , and M. Kobayashi . 2013. “Function and Characteristics of PINK1 in Mitochondria.” Oxidative Medicine and Cellular Longevity 2013: 601587. 23533695 10.1155/2013/601587 PMC3600171

[cph470029-bib-0053] Mettler, J. A. , and L. Griffin . 2016. “Muscular Endurance Training and Motor Unit Firing Patterns During Fatigue.” Experimental Brain Research 234: 267–276. 26449966 10.1007/s00221-015-4455-x

[cph470029-bib-0054] Molkov, Y. I. , G. Yu , J. Ausborn , J. Bouvier , S. M. Danner , and I. A. Rybak . 2024. “Sensory Feedback and Central Neuronal Interactions in Mouse Locomotion.” Royal Society Open Science 11: 240207. 39169962 10.1098/rsos.240207 PMC11335407

[cph470029-bib-0055] Nokia, M. S. , S. Lensu , J. P. Ahtiainen , et al. 2016. “Physical Exercise Increases Adult Hippocampal Neurogenesis in Male Rats Provided It Is Aerobic and Sustained.” Journal of Physiology 594: 1855–1873. 26844666 10.1113/JP271552 PMC4818598

[cph470029-bib-0056] Nulali, J. , M. Zhan , K. Zhang , P. Tu , Y. Liu , and H. Song . 2022. “Osteoglycin: An ECM Factor Regulating Fibrosis and Tumorigenesis.” Biomolecules 12: 1674. 10.3390/biom12111674. 36421687 PMC9687868

[cph470029-bib-0057] Pedersen, B. K. , and B. Saltin . 2006. “Evidence for Prescribing Exercise as Therapy in Chronic Disease.” Scandinavian Journal of Medicine & Science in Sports 16, no. Suppl 1: 3–63. 16451303 10.1111/j.1600-0838.2006.00520.x

[cph470029-bib-0058] Piccirillo, R. 2019. “Exercise‐Induced Myokines With Therapeutic Potential for Muscle Wasting.” Frontiers in Physiology 10: 287. 30984014 10.3389/fphys.2019.00287 PMC6449478

[cph470029-bib-0059] Popov, D. V. , E. A. Lysenko , R. O. Bokov , et al. 2018. “Effect of Aerobic Training on Baseline Expression of Signaling and Respiratory Proteins in Human Skeletal Muscle.” Physiological Reports 6: e13868. 30198217 10.14814/phy2.13868 PMC6129775

[cph470029-bib-0060] Rahimi, O. , A. C. Melo , B. Westwood , R. D. M. Grier , E. A. Tallant , and P. E. Gallagher . 2022. “Angiotensin‐(1‐7) Reduces Doxorubicin‐Induced Aortic Arch Dysfunction in Male and Female Juvenile Sprague Dawley Rats Through Pleiotropic Mechanisms.” Peptides 152: 170784. 35288251 10.1016/j.peptides.2022.170784

[cph470029-bib-0061] Rivas, D. A. , S. J. Lessard , M. Saito , et al. 2011. “Low Intrinsic Running Capacity Is Associated With Reduced Skeletal Muscle Substrate Oxidation and Lower Mitochondrial Content in White Skeletal Muscle.” American Journal of Physiology Regulatory, Integrative and Comparative Physiology 300: R835–R843. 21270346 10.1152/ajpregu.00659.2010 PMC3075075

[cph470029-bib-0062] Ross, R. , B. H. Goodpaster , L. G. Koch , et al. 2019. “Precision Exercise Medicine: Understanding Exercise Response Variability.” British Journal of Sports Medicine 53: 1141–1153. 30862704 10.1136/bjsports-2018-100328 PMC6818669

[cph470029-bib-0063] Rossignol, S. , R. Dubuc , and J.‐P. Gossard . 2006. “Dynamic Sensorimotor Interactions in Locomotion.” Physiological Reviews 86: 89–154. 16371596 10.1152/physrev.00028.2005

[cph470029-bib-0064] Roy, R. R. , D. L. Hutchison , D. J. Pierotti , J. A. Hodgson , and V. R. Edgerton . 1991. “EMG Patterns of Rat Ankle Extensors and Flexors During Treadmill Locomotion and Swimming.” Journal of Applied Physiology 70: 2522–2529. 1885445 10.1152/jappl.1991.70.6.2522

[cph470029-bib-0065] Sandri, M. , C. Sandri , A. Gilbert , et al. 2004. “Foxo Transcription Factors Induce the Atrophy‐Related Ubiquitin Ligase Atrogin‐1 and Cause Skeletal Muscle Atrophy.” Cell 117: 399–412. 15109499 10.1016/s0092-8674(04)00400-3 PMC3619734

[cph470029-bib-0066] Smith, J. A. B. , K. A. Murach , K. A. Dyar , and J. R. Zierath . 2023. “Exercise Metabolism and Adaptation in Skeletal Muscle.” Nature Reviews Molecular Cell Biology 24: 607–632. 37225892 10.1038/s41580-023-00606-x PMC10527431

[cph470029-bib-0067] Steele, J. , J. Fisher , M. Skivington , et al. 2017. “A Higher Effort‐Based Paradigm in Physical Activity and Exercise for Public Health: Making the Case for a Greater Emphasis on Resistance Training.” BMC Public Health 17: 300. 28381272 10.1186/s12889-017-4209-8 PMC5382466

[cph470029-bib-0068] Szaroszyk, M. , B. Kattih , A. Martin‐Garrido , et al. 2022. “Skeletal Muscle Derived Musclin Protects the Heart During Pathological Overload.” Nature Communications 13: 149. 10.1038/s41467-021-27634-5 PMC8748430 35013221

[cph470029-bib-0069] Tharmaratnam, T. , R. A. Civitarese , T. Tabobondung , and T. A. Tabobondung . 2017. “Exercise Becomes Brain: Sustained Aerobic Exercise Enhances Hippocampal Neurogenesis.” Journal of Physiology 595: 7–8. 28035680 10.1113/JP272761 PMC5199726

[cph470029-bib-0070] Thompson, J.‐L. M. , D. W. D. West , T. M. Doering , et al. 2022. “Effect of Short‐Term Hindlimb Immobilization on Skeletal Muscle Atrophy and the Transcriptome in a Low Compared With High Responder to Endurance Training Model.” PLoS One 17: e0261723. 35025912 10.1371/journal.pone.0261723 PMC8757917

[cph470029-bib-0071] Thyfault, J. P. , R. S. Rector , G. M. Uptergrove , et al. 2009. “Rats Selectively Bred for Low Aerobic Capacity Have Reduced Hepatic Mitochondrial Oxidative Capacity and Susceptibility to Hepatic Steatosis and Injury.” Journal of Physiology 587: 1805–1816. 19237421 10.1113/jphysiol.2009.169060 PMC2683966

[cph470029-bib-0072] Tieland, M. , I. Trouwborst , and B. C. Clark . 2018. “Skeletal Muscle Performance and Ageing.” Journal of Cachexia, Sarcopenia and Muscle 9: 3–19. 29151281 10.1002/jcsm.12238 PMC5803609

[cph470029-bib-0073] Vanderheyden, W. , M. Kehoe , G. Vanini , S. L. Britton , and L. G. Koch . 2021. “Rat Models for Low and High Adaptive Response to Exercise Differ for Stress‐Related Memory and Anxiety.” Physiological Reports 9: e14716. 33619911 10.14814/phy2.14716 PMC7900769

[cph470029-bib-0074] West, D. W. D. , T. M. Doering , J.‐L. M. Thompson , et al. 2021. “Low Responders to Endurance Training Exhibit Impaired Hypertrophy and Divergent Biological Process Responses in Rat Skeletal Muscle.” Experimental Physiology 106: 714–725. 33486778 10.1113/EP089301 PMC8686756

[cph470029-bib-0075] Wilkinson, D. J. , M. Piasecki , and P. J. Atherton . 2018. “The Age‐Related Loss of Skeletal Muscle Mass and Function: Measurement and Physiology of Muscle Fibre Atrophy and Muscle Fibre Loss in Humans.” Ageing Research Reviews 47: 123–132. 30048806 10.1016/j.arr.2018.07.005 PMC6202460

[cph470029-bib-0076] Wisløff, U. , A. Bye , T. Stølen , et al. 2015. “Blunted Cardiomyocyte Remodeling Response in Exercise‐Resistant Rats.” Journal of the American College of Cardiology 65: 1378–1380. 25835453 10.1016/j.jacc.2015.01.041

[cph470029-bib-0077] Wisløff, U. , S. M. Najjar , O. Ellingsen , et al. 2005. “Cardiovascular Risk Factors Emerge After Artificial Selection for Low Aerobic Capacity.” Science 307: 418–420. 15662013 10.1126/science.1108177

[cph470029-bib-0078] Xu, J. , R. Li , B. Workeneh , Y. Dong , X. Wang , and Z. Hu . 2012. “Transcription Factor FoxO1, the Dominant Mediator of Muscle Wasting in Chronic Kidney Disease, Is Inhibited by microRNA‐486.” Kidney International 82: 401–411. 22475820 10.1038/ki.2012.84 PMC3393843

[cph470029-bib-0079] Zhang, D. , X. Liu , Y. Liu , et al. 2017. “Leisure‐Time Physical Activity and Incident Metabolic Syndrome: A Systematic Review and Dose‐Response Meta‐Analysis of Cohort Studies.” Metabolism 75: 36–44. 28927737 10.1016/j.metabol.2017.08.001

[cph470029-bib-0080] Zhang, X. , D. A. Englund , Z. Aversa , S. K. Jachim , T. A. White , and N. K. LeBrasseur . 2022. “Exercise Counters the Age‐Related Accumulation of Senescent Cells.” Exercise and Sport Sciences Reviews 50: 213–221. 35776782 10.1249/JES.0000000000000302 PMC9680689

[cph470029-bib-0081] Zuo, Z. , M.‐H. Li , X.‐H. Zheng , W.‐M. Yao , H. Wang , and X.‐L. Li . 2022. “Elevated Plasma Levels of Osteoglycin in Cardiovascular Patients: A Systematic Review and Meta‐Analysis.” Annals of Palliative Medicine 11: 498–505. 35249327 10.21037/apm-22-104

